# Comparison and combination of gamified neurofeedback training and general behavioral training

**DOI:** 10.1371/journal.pone.0278762

**Published:** 2022-12-02

**Authors:** Ming Chang, Yusuke Yokota, Hideyuki Ando, Taro Maeda, Yasushi Naruse

**Affiliations:** 1 Center for Information and Neural Networks (CiNet), National Institute of Information and Communications Technology and Osaka University, Kobe, Hyougo, Japan; 2 Graduate School of Information Science and Technology, Osaka University, Suita, Osaka, Japan; 3 Osaka University of Arts, Osaka, Japan; Union University, SERBIA

## Abstract

With the rapid development of the international community, foreign language learning has become increasingly important. Listening training is a particularly important component of foreign language learning. The most difficult aspect of listening training is the development of speech discrimination ability, which is crucial to speech perception. General behavioral training requires a substantial amount of time and attention. To address this, we previously developed a neurofeedback (NF) training system that enables unconscious learning of auditory discrimination. However, to our knowledge, no studies have compared NF training and general behavioral training. In the present study, we compared the learning effects of NF training, general behavioral training, and a combination of both strategies. Specifically, we developed a gamified and adapted NF training of auditory discrimination. We found that both NF training and general behavioral training enhanced behavioral performance, whereas only NF training elicited significant changes in brain activity. Furthermore, the participants that used both training methods exhibited the largest improvement in behavioral performance. This indicates that the combined use of NF and general behavioral training methods may be optimal for enhancing auditory discrimination ability when learning foreign languages.

## Introduction

English is the dominant language of international communication in the globalized world. Because of rapid growth in the demand for English proficiency in various fields, the improvement of English language skills has become an important issue. Unfortunately, many Japanese individuals struggle with English after years of study. One important reason is the difficulty in distinguishing speech sounds that are not part of the Japanese language (e.g., /l/ and /r/) [1–5]. This ability is very important for speech perception and for improving English proficiency. However, because one phonological category in Japanese subsumes two different ones in English, it is difficult for Japanese-speaking adults to perceive this difference. As a result, learning to distinguish these foreign sounds requires a long period of practice.

In some previous studies, a general behavioral training paradigm was used to train participants to distinguish between sounds in English. Specifically, they used a two-alternative forced-choice identification task in which participants received binary feedback (correct/incorrect) regarding the accuracy of their responses. For example, learners listened to words that included the phonemes /l/ or /r/, and then discriminated between the words by pressing response buttons. Immediate feedback regarding response accuracy was given to the learners [6, 7]. Recent studies have focused on subconscious perceptual learning using neurofeedback (NF) technology [8, 9]. NF training is a non-invasive method that uses electroencephalography (EEG) to enable people to ’watch’ their own brain activity in real time and to control or adjust function in predetermined brain regions [10, 11]. Moreover, recent studies indicate that brain computer interface (BCI)-based applications could successfully regulate users’ cognitive abilities like memory, attention, communication and emotion or entertainment during training and rehabilitation [12–16]. BCI-based neurofeedback training involves learning structures in the brain that require users to achieve certain goals by controlling their brain signals through mental imagery strategies. In order to increase BCI reliability to a higher level, a novel moving sound spatial auditory BCI paradigm was presented, which improved single trial event-related potential (ERP) response classification results [17]. However, there have been no studies using BCI techniques to improve speech sounds discrimination ability.

We previously developed an NF system designed to help users improve their ability to distinguish speech sounds that are not part of the Japanese language [18, 19]. We used mismatch negativity (MMN), which is a component of the auditory ERP elicited while learners listen to sounds. The MMN response can be caused by any discernible change in an auditory stimulus. The characteristics of MMN can also reflect the accuracy of sound discrimination [20, 21]. Changes in the amplitude of the MMN elicited by sound stimuli were visualized as changes in the size of a visual stimulus presented to learners. Participants were asked to try to increase the size of the circle in the visual stimulus as much as they could to improve their brain activity [18, 19, 22, 23]. Because MMN can be elicited without subjectively requiring the listener to pay attention to the auditory stimuli [24, 25], this NF method does not require the learner to focus on the auditory stimuli or be aware of the learning process. Thus, this type of strategy has the potential to bring about a drastic change in the concept of learning itself.

Although our previous study [18, 19] confirmed that a MMN NF training method enhanced behavioral performance, it has not yet been compared with general behavioral training. Thus, whether NF training is more effective than general behavior training is still unknown. Furthermore, a combination of the two approaches could hinder or enhance learning. According to participant interviews conducted after NF training in our previous experiment, the auditory discrimination training method was considered very boring because the visual stimulus lacked diversification. Therefore, we applied gamification to the NF training in the present study.

Here, we designed and implemented a gamified and adapted NF training with 3 game scenarios to choose from. We anticipated that this would reduce participant boredom during training by increasing variation. In the NF training sessions, MMN amplitudes were presented to participants as visual feedback regarding discrimination accuracy, which functioned as a continuous cue for learning. The present study had two purposes. The first was to compare NF training and general behavioral training in terms of behavioral performance and neural activity. The second was to investigate whether using the two training methods together would have a greater learning effect than when they were applied individually. We hypothesized that there would be no conflict between these two methods, and that using them together would enhance the learning effect. To test this, we divided the participants into four groups according to the experimental conditions they participated in (see details in the section of participants).

## Methods

**Fig 1:**
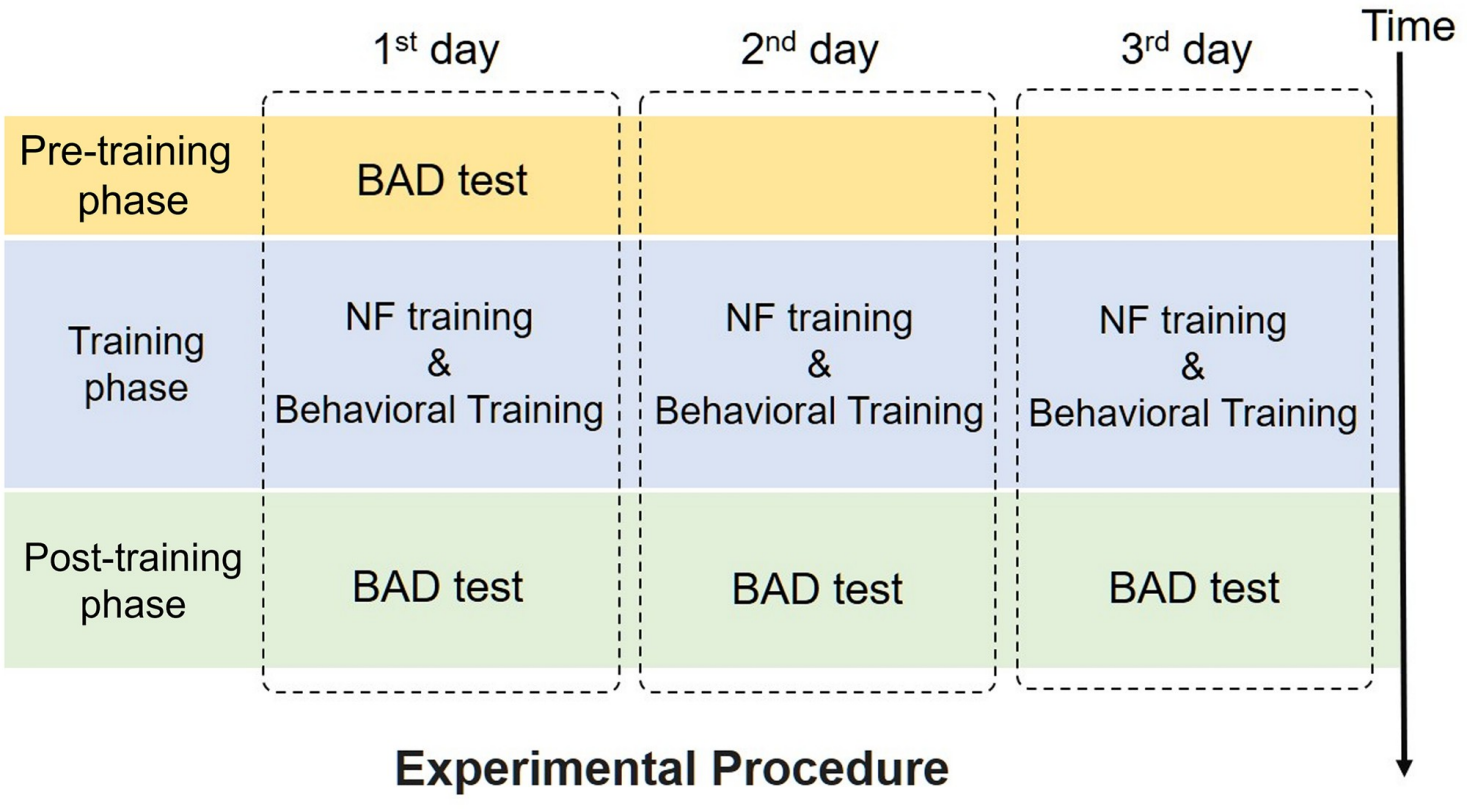
Overall study procedure.

The experiment had a pre-training and post-training test procedure, similar to that in previous studies [18, 19]. This process consisted of a pre-training phase, a training phase, and a post-training phase (Fig 1). A behavioral auditory discrimination (BAD) test was conducted in the pre-training and post-training phases. In the training phase, each participant was trained to learn a pair of word sounds on 3 separate days. The interval between training on each of the 3 days was more than 24 hours, and the entire training phase was completed within 5 days. This training phase was similar to that in our previous study [19].

### Participants

We used an independent company to recruit 32 native Japanese speakers (16 men, 16 women; age range: 20–49 years). None of the participants had lived abroad. They reported that they had similar educational experience in terms of learning English. Specifically, they started studying English at about age 12 in Japanese schools, and were mostly exposed to English in the classroom. All participants had normal hearing and normal or corrected-to-normal vision, and no participants had hearing or speech disorders.

**Fig 2:**
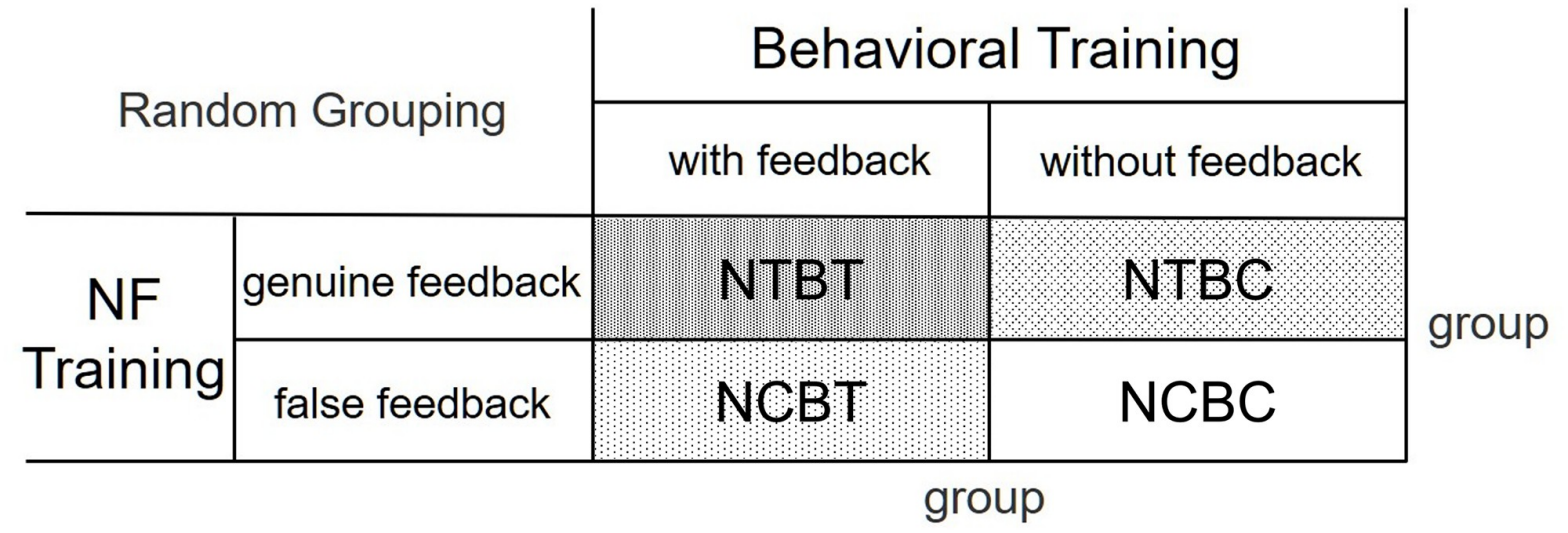
Participant grouping.

Although the participants were instructed to ignore the auditory stimulus during training, they continued to listen to the auditory stimulus, which may have induced auditory discrimination learning unconsciously. To eliminate these possibilities, a control condition that was identical to the experimental environment including auditory and visual stimuli used for the training condition was required. Therefore, the participants were randomly separated into the following four groups according to their exposure to NF training and behavioral training (Fig 2): The NTBT group (participation in NF training condition with genuine feedback and behavioral training condition with genuine feedback), NTBC group (participation in NF training condition with genuine feedback and behavioral control condition without feedback), NCBT group (participation in NF control condition with false feedback and behavioral training condition with genuine feedback), and NCBC group (participation in NF control condition with false feedback and behavioral control condition without feedback). The composition of each group was the same (4 men, 4 women). None of the participants were aware of their group identity, which avoided affecting their subjective willingness to learn.

Before the experiment, we explained the details of the procedure to the participants and asked them to provide informed written consent. All experimental procedures were carried out in accordance with the ethical standards set out in the Declaration of Helsinki and approved by the Ethical Committee for Human and Animal Research of the National Institute of Information and Communications Technology of Japan.

### Stimuli

The auditory stimuli used in the experiment were synthesized sounds, “light” (/laɪt/) and “right” (/raɪt/), created using custom sound editing software called “TTSEditor” (National Institute of Information and Communications Technology, Tokyo, Japan) [26]. The duration of the two word sounds was 400 ms, including a 10 ms rise and fall time. To ensure that the sound stimulation was within the auditory comfort range of each participant, the earphone volume was calibrated before the experiment.

### Behavioral auditory discrimination test

We used a two-alternative forced choice task to assess behavioral auditory discrimination ability. In this test, one of four stimulus combinations made up of two word sounds was presented (the four stimulus permutations were “light/right,” “light/light,” “right/right,” and “right/light”). The presentation order was random and counterbalanced across blocks (all combinations were presented in equal proportions). The stimulus onset asynchrony of the two stimuli in each combination was 800 ms. The participants were tested individually, and the stimuli were presented at a comfortable listening level over headphones. The participants were asked to press the “different” button if they judged two stimuli in one trial to be different, or press the “same” button if the two stimuli in one trial were considered to be the same.

We presented 96 trials in two blocks. Before the experimental trials, the participants were familiarized with the task using word stimuli that were different from those used during the experiment. The participants did not receive feedback from the experimenters during the practice sessions or experiment.

### Behavioral training

**Fig 3:**
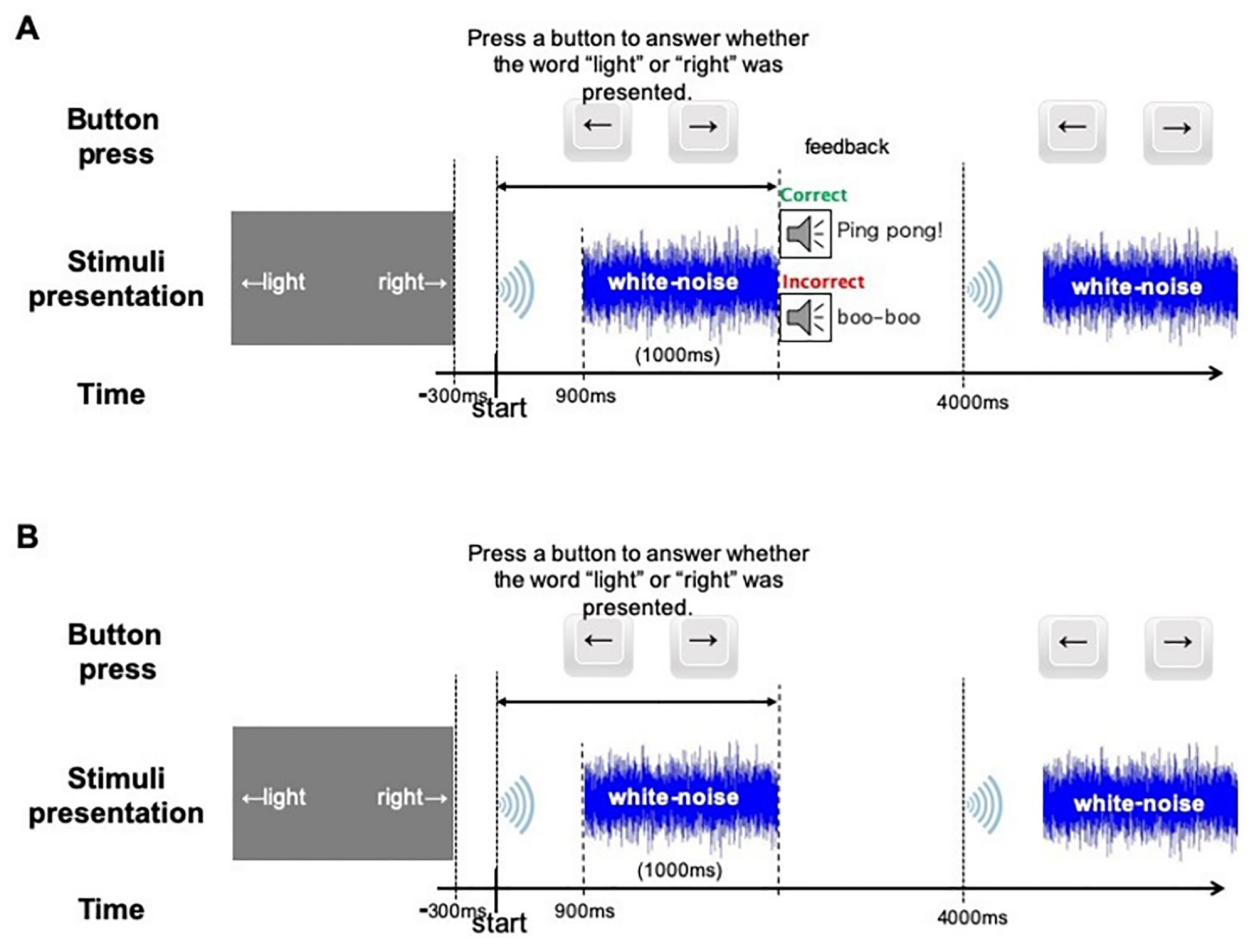
Schematic drawing of behavioral training with feedback (A) and without feedback (B).

We used a two-alternative forced choice word pair identification task for behavioral training in the training phase (Fig 3). At the start of each trial, 300 ms before the auditory stimulus, the standard English orthographies for the “light/right” pair were presented on the computer monitor. One word appeared in the middle left of the screen, and the other word appeared in the middle right. The word sound was then presented and participants identified the word sound by pressing a button on a keyboard. Participants were given 1.9 s to respond by pressing the “←” or “→” button. They were instructed to press the “←” button if they judged the spoken word to be the word on the left of the screen, and to press the “→” button if the word was considered to be the word on the right of the screen. During behavioral training with genuine feedback, after the participants responded, audible feedback was given to indicate whether the response was correct (Fig 3A). A chime meant the response was correct, and a buzzer meant the response was incorrect. All 96 trials were presented in two blocks. To begin, the participants were familiarized with the task using word stimuli that were different from those used in the experiment. During behavioral training without feedback, the participants received the same stimuli and instructions, but no feedback was presented after they pressed the button (Fig 3B).

### Data acquisition

EEG data were acquired using wireless EEG recording device (Polymate Mini AP108; Miyuki Giken Co., Ltd., Tokyo, Japan). The electrodes were located at positions FPz according to the 10–20 international electrode placement system, and placed on the right and left mastoids as the reference and ground electrodes, respectively. In addition, one electrode was placed on the upper-outer edge of the left eye to assess electro-ocular (EOG) activity. EEG data were continuously sampled at a frequency of 500 Hz and sent to a computer via Bluetooth.

For EEG acquisition, we used only one channel to calculate the MMN. This was because FPz is located close to Fz, and we found it to be feasible for measuring MMN in our previous study [19]. Although collecting signal data from a greater number of channels may lead to more accurate results, the convenience and practicality of our method will be reduced when the number of channels is increased, which would affect the application of our method in daily life. Specifically, the amount of time required to set up the device would increase, as would the data processing period.

### Signal processing

The digitized data from the EEG amplifier was subjected to signal processing using a custom program written in visual C#. Online, a 0.1–35 Hz band-pass filter was applied to the EEG and EOG signals. The EEG and EOG epochs associated with individual trials were extracted for the period 100 ms before to 500 ms after the onset of the auditory stimulus. The 100 ms pre-stimulus interval preceding the onset of the auditory stimulus was used as a baseline to measure the amplitude. Epochs with amplitudes for which the absolute value exceeded 40 μV were removed as artefactual epochs.

NF training to improve auditory ability requires the enhancement of MMN amplitudes. We calculated MMN amplitudes as the absolute value of the most negative peak in the difference waveform at 100–250 ms from the stimulus onset. The difference waveform was obtained by subtracting the average waveform of ERPs elicited by standard stimuli from the average waveform of ERPs elicited by deviant stimuli.

To perform NF training, we needed to calculate the MMN amplitude in real time. Thus, we used a calculation method that enabled us to update the values of the MMN amplitude in real time. We first calculated the average MMN amplitude for the first 20 trials (deviant:standard = 1:4), and then recalculated the MMN amplitude starting with the 21^th^ trial, using the response in each new trial to replace that from the oldest trial. If the new trial contained the standard stimulus, it would replace the oldest standard trial. Similarly, if the new trial contained the deviant stimulus, it would replace the oldest deviant trial. Therefore, the MMN amplitudes were recalculated and updated every 800 ms as the trials progressed. In addition, before the NF training began, we set a maximal MMN for the participants to strive to achieve. We used an auditory oddball paradigm to calculate the maximal MMN amplitude for each participant. We used 1000 Hz and 2000 Hz tones as the standard stimulus and deviant stimulus, respectively, in the auditory oddball paradigm. The maximal MMN amplitude was the absolute value of the MMN amplitude elicited by 1000 Hz and 2000 Hz tones.

### NF training game design

**Fig 4:**
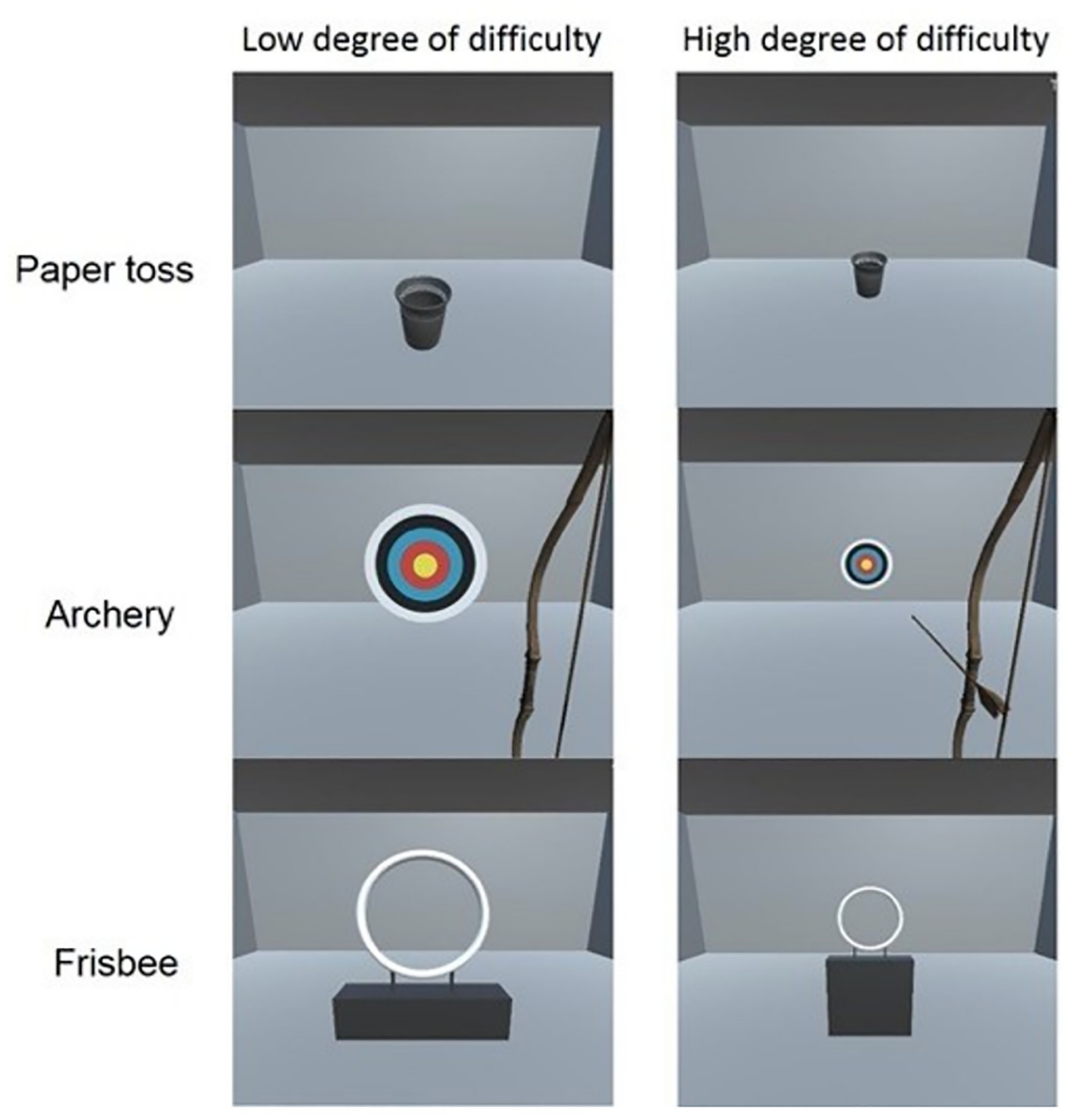
The 3 different BCI games for NF training.

The program of NF training game for this project was implemented in C#. As the MMN sequence required “oddball” stimuli, the selected auditory stimuli needed to be distinct from other stimuli in the game. Accordingly, we needed to integrate the oddball stimuli into the game environment in a creative and unobtrusive way, and design the game to be simpler than a standard computer game. Taking into account the individual preferences of the participants, we provided them with 3 scenarios. We anticipated that this variation would reduce boredom during training. To prevent excessive eye movement during the game, we needed the participants to focus on a single point. Accordingly, we designed games in which the player aimed at a target point. In our previous study, we used the paper toss game for NF training [27], and found a significant training effect. Thus, we added two new game scenarios that had the same paradigm as that in the paper toss game, namely, an archery game and a Frisbee game (Fig 4). We chose these because they are both target-based games, and because we anticipated that incorporating elements of sports and entertainment would make the trials less boring. As indicated in Fig 4, in the paper toss game, participants were instructed to try to toss a spitball into a garbage can. In the archery game, participants were instructed to try to shoot an arrow at a target. In the Frisbee game, the participants were instructed to try to throw a Frisbee through a target ring. In all games, the participants were asked to ignore the sounds and only focus on the visual feedback.

In each game scenario, the participants received one point if they succeeded in achieving the goal. For example, in the paper toss game, whether the spitball could be thrown into the target (garbage can) depended on whether the amplitude of the MMN was greater than the threshold. In each game scenario, there were four stages with different threshold values. The threshold increased as the stage progressed, making the game more and more difficult. The threshold values were 20% of the maximal MMN in the 1^st^ stage, 40% of the maximal MMN in the 2^nd^ stage, 50% of the maximal MMN in the 3^rd^ stage, and 60% of the maximal MMN in the 4^th^ stage. The threshold value was visualized as the distance from the starting point to the garbage can in the paper toss game, the size of the target in the archery game, and the size of the target ring in the Frisbee game, respectively (Fig 4). When the participants received points in more than half of the games in a stage, they would clear the stage and move on to the next one. If a participant could not clear a stage, they continued to play the game at that stage.

Each participant performed 12 sessions (4 sessions per game) on each training day, and each session was composed of a sequence of 300 sounds (0.8 s × 300 = 240 s). The 3 games were not interspersed, and a new game was presented only after the participant had completed 4 sessions of one game. The order of the 3 games was random and was counterbalanced across participants and days. During the training period, there was a short break between sessions.

Although the participants in the NF control groups (NCBT and NCBC groups) received the same stimuli and played the same NF training games, the MMN responses that determined the game progress were not their own, but instead those of participants in the NF training groups (NTBT and NTBC groups). Thus, there was no correlation between game progress and MMN responses in the NF control groups.

## Result

**Fig 5:**
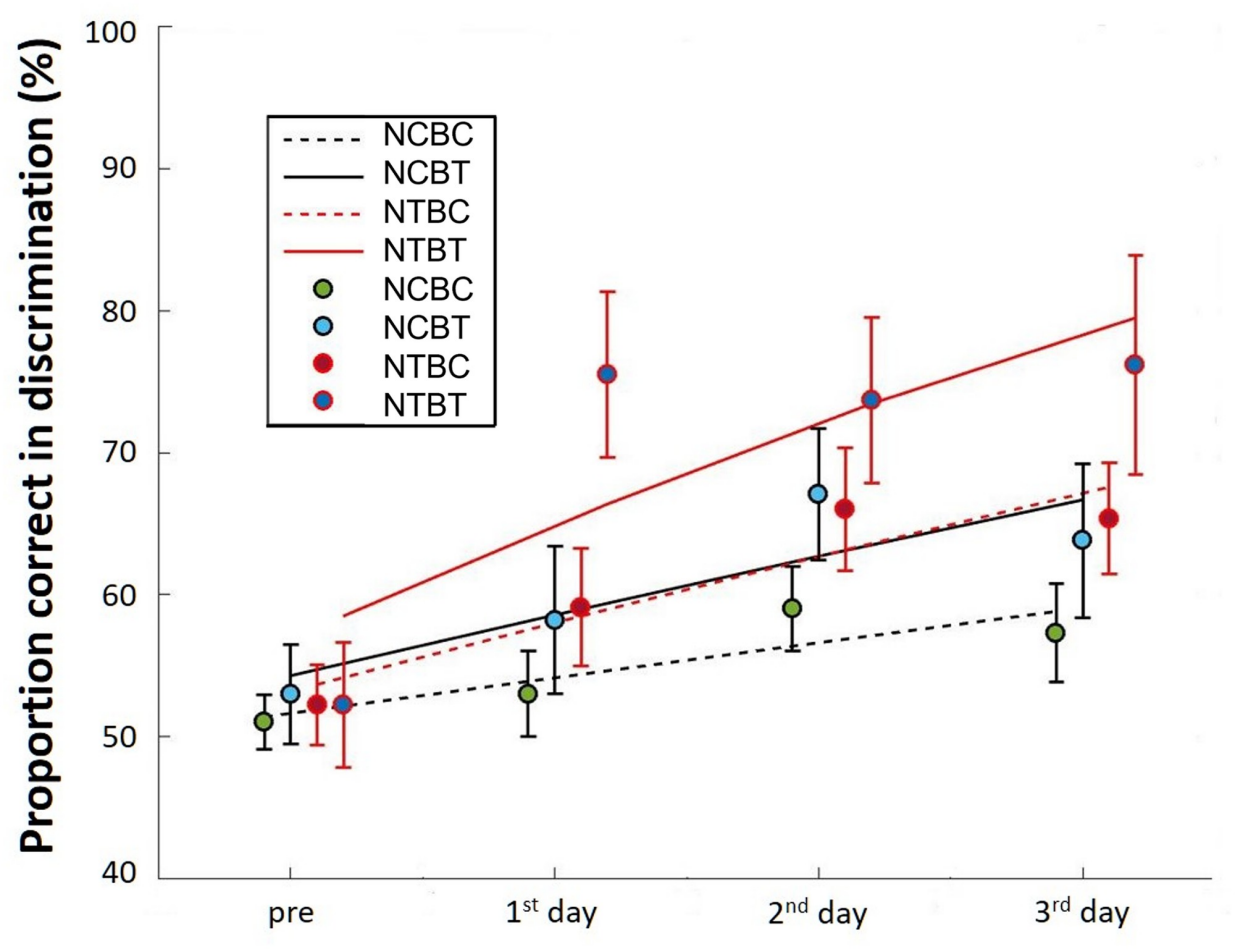
Performance in the BAD test. Average proportion of correct responses and logit models for pre-training test and post-training test data for the NCBC group (black dashed line), NCBT group (black solid line), NTBC group (red dashed line), and NTBT group (red solid line) in the discrimination test on each training day for two sounds. Error bars represent the standard error of the mean (SEM).

To assess improvements in auditory perception, we compared the probability of correct responses in the BAD test in the pre-training test and post-training test phases (Fig 5). Here, we choose to use logistic regression to analyze the differences between outcomes before versus after training because the outcomes are dichotomous. We used R-Studio (version 4.4.1) to process and statistically analyze the BAD test data. The probability of correct responses was analyzed using a generalized linear mixed-effects model (GLMM) with the lmer function in the lme4 package [28] and dummy coding.

In this study, the maximal GLMM model described in Wilkinson notation was as follows: cbind (the number of correct responses, the number of incorrect responses) ~ Group * Day + (1 | participants). This model indicates that the probability of correct responses was modeled using Group (NTBT vs. NTBC vs. NCBT vs. NCBC), Day (pre vs. 1^st^ day vs. 2^nd^ day vs. 3^rd^ day), and interactions as fixed effects. A participant difference was defined as a random intercept (Participants). The model for estimating the probability of correct responses in the BAD test is summarized in Table 1.

**Table 1:** Probability of correct responses in the BAD test.

Variable	Estimate	SE	z-value	p-value
Intercept (NTBT, pre)	0.0892	0.0903	0.987	0.324
Group (NTBC)	-0.0000	0.1025	0.000	0.999
Group (NCBT)	0.0315	0.1025	0.307	0.759
Group (NCBC)	-0.0472	0.1110	-0.461	0.645
1^st^ day	1.0430	0.1181	9.399	< 0.001 ***
2^nd^ day	0.9467	0.1095	8.643	< 0.001 ***
3^rd^ day	1.0790	0.1116	9.669	< 0.001 ***
Group (NTBC): 1^st^ day	-0.7617	0.1516	-5.024	< 0.001 ***
Group (NCBT): 1^st^ day	-0.8309	0.1515	-5.484	< 0.001 ***
Group (NCBC): 1^st^ day	-0.9646	0.1511	-6.386	< 0.001 ***
Group (NTBC): 2^nd^ day	-0.3681	0.1519	-2.423	0.015 *
Group (NCBT): 2^nd^ day	-0.3526	0.1522	-2.316	0.021 *
Group (NCBC): 2^nd^ day	-0.6232	0.1505	-4.141	< 0.001 ***
Group (NTBC): 3^rd^ day	-0.5295	0.1532	-3.455	< 0.001 ***
Group (NCBT): 3^rd^ day	-0.6297	0.1529	-4.119	< 0.001 ***
Group (NCBC): 3^rd^ day	-0.8255	0.1518	-5.438	< 0.001 ***
**Variance Components**	**SD**	**Goodness of fit**		
**Participants**	**0.1525**	**Log likelihood**	**-817.0**	

We used type III Wald χ2 tests to determine the significance of the fixed effects based on car packages [29]. Fig 5 shows the proportion of correct response outcomes for each level of group and day. An analysis of variance based on mixed logistic regression indicated a statistically significant effect of Group (χ2 (3) = 144.85, p < 0.001), Day (χ2 (3) = 173.33, p < 0.001), and a statistically significant Group×Day interaction (χ2 (9) = 58.39, p < 0.001) (Table 2).

**Table 2:** Wald χ2 test results in the GLMM for Groups and Days.

Fixed effects	*χ* ^*2*^	*Df*	*P*
**Group**	144.85	3	< 0.001 ***
**Day**	173.33	3	< 0.001 ***
**Group:Day**	58.39	9	< 0.001 ***

df, degrees of freedom.

***p < 0.001.

For the post-hoc tests, we performed multiple comparisons of Groups and Days based on the fitted GLMM, using the Kenward-Roger method and Tukey’s HSD test to correct p values for pairwise comparisons. All post-hoc tests were performed using the emmeans packages [30]. For pairwise comparisons at the group level (Table 3), we observed no significant differences between the groups on the first day. However, from the 1^st^ day after training, the NTBT group showed a significant improvement compared with the other three groups, and from the 2^nd^ day after training, the NTBC and NCBT groups showed a significant improvement compared with the NCBC group. We found no significant differences between the NTBC group and NCBT group. For the pairwise comparisons at the days level (Table 4), compared with the pre-test, only the NTBT and NTBC groups showed significant improvements on the 1^st^ day. Although all four groups showed significant improvements on the 2^nd^ day after training, all groups except for the NCBC group showed significant improvements after completing all training sessions.

**Table 3:** Summaries of the post-hoc tests for pairwise comparisons of Groups on each day.

Group contrast	Pre		1^st^ day		2^nd^ day		3^rd^ day	
	OR	*p*	OR	*p*	OR	*p*	OR	*p*
**NTBT—NTBC**	1.000	1.0000	2.142	**<.0001**	1.445	**0.0057**	1.698	**<.0001**
**NTBT—NCBT**	0.969	0.9899	2.224	**<.0001**	1.379	**0.0225**	1.819	**<.0001**
**NTBT—NCBC**	1.048	0.9675	2.751	**<.0001**	1.955	**<.0001**	2.393	**<.0001**
**NTBC—NCBT**	0.969	0.9899	1.039	0.9836	0.954	0.9727	1.071	0.9184
**NTBC—NCBC**	1.048	0.9675	1.284	0.0731	1.353	**0.0226**	1.409	**0.0063**
**NCBT—NCBC**	1.082	0.8688	1.237	0.1667	1.418	**0.0057**	1.316	**0.0442**

OR, odds ratio.

**Table 4:** Summaries of the post-hoc tests for pairwise comparisons of Days on each group.

Day contrast	NTBT		NTBC		NCBT		NCBC	
	OR	*p*	OR	*p*	OR	*p*	OR	*p*
**pre - 1**^**st**^ **day**	0.352	**<.0001**	0.755	**0.0324**	0.809	0.1667	0.924	0.8688
**pre - 2**^**nd**^ **day**	0.388	**<.0001**	0.561	**<.0001**	0.552	**<.0001**	0.724	**0.0093**
**pre - 3**^**rd**^ **day**	0.340	**<.0001**	0.577	**<.0001**	0.638	**0.0001**	0.776	0.0658
**1**^**st**^ **day - 2**^**nd**^ **day**	1.101	0.8439	0.743	**0.0264**	0.683	**0.0019**	0.783	0.0830
**1**^**st**^ **day - 3**^**rd**^ **day**	0.965	0.9907	0.765	0.0552	0.789	0.1090	0.840	0.3251
**2**^**nd**^ **day - 3**^**rd**^ **day**	0.876	0.6766	1.029	0.9932	1.156	0.5345	1.072	0.9069

OR, odds ratio.

**Fig 6:**
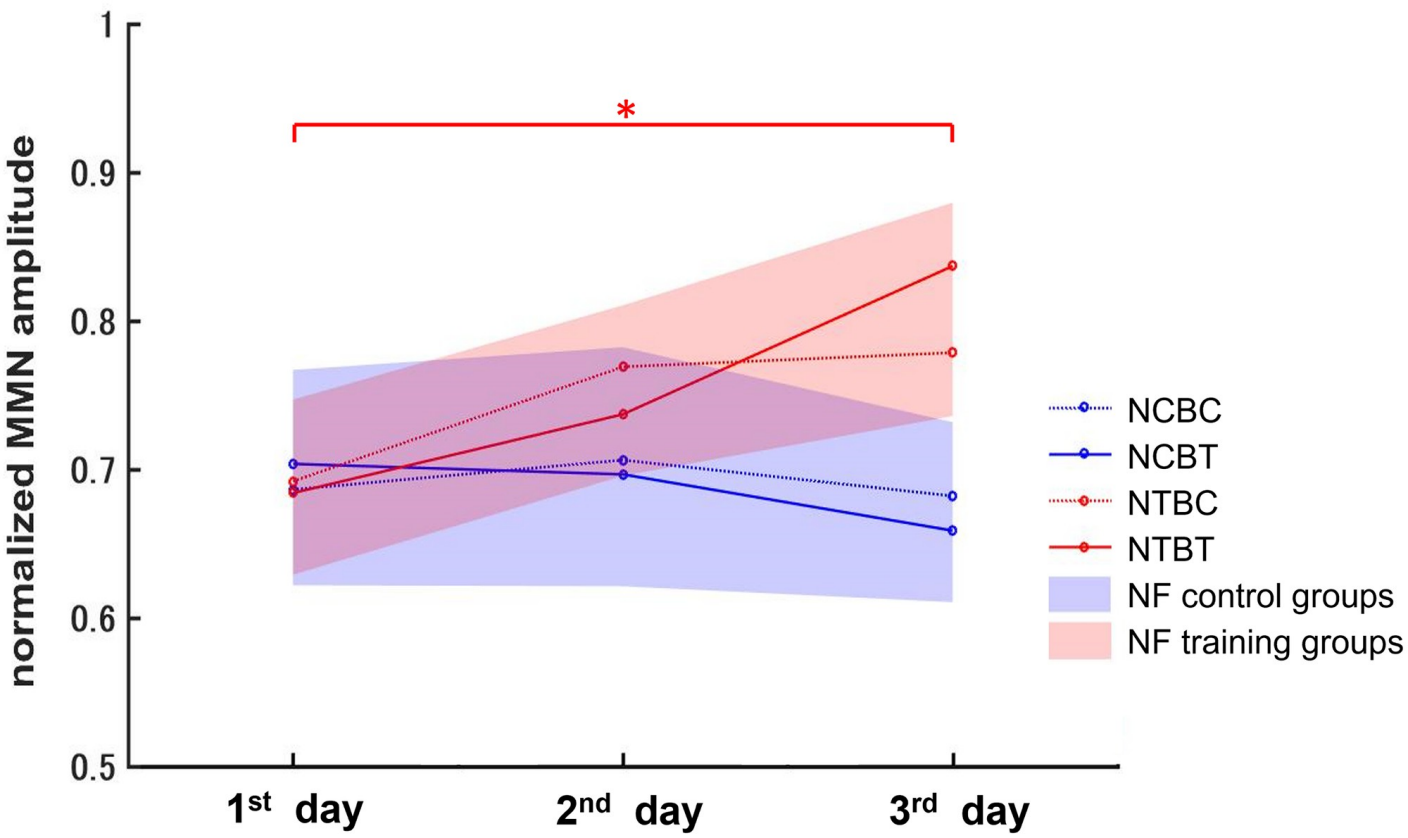
Results of changes in neural activity. Normalized values of average MMN amplitudes for all participants on each training day in the four groups (NCBC group: blue dotted line; NCBT group: blue solid line; NTBC group: red dotted line; NTBT group: red solid line). The range of the blue line width represents the SEM for the group without NF training, and the red width range represents the SEM for the group with NF training; *p < 0.05.

**Fig 7:**
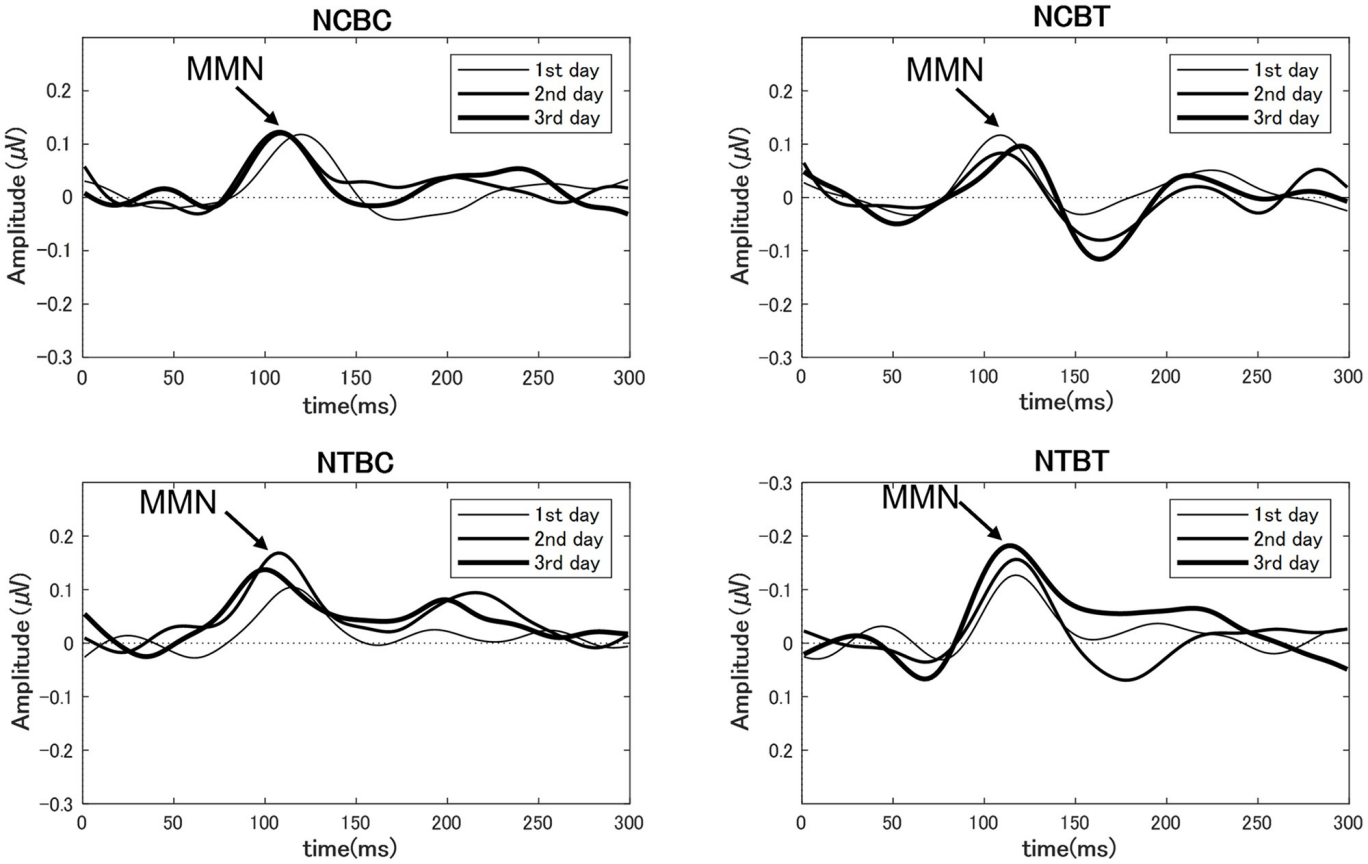
The group grand average MMN responses on each training day in four groups respectively.

We also assessed whether neural activity changed in the four groups. Using the EEG data collected on each training day, we calculated the average MMN amplitudes for all participants and normalized the values of the MMN amplitudes based on the maximal MMN measured before training on the first training day. Fig 6 shows the normalized group grand average MMN responses on each training day in the four groups. Normalized MMN amplitudes were submitted to a repeated measures multivariate analysis of variance with NF training (genuine feedback vs. false feedback), Behavioral training (with feedback vs. without feedback), and Training stage (1st day vs. 2nd day vs. 3rd day) as factors. The analysis of variance indicated a significant interaction between NF training and Training stage (F (2, 54) = 4.26, p < 0.05). We found no significant effects for Behavioral training or other interactions. We performed tests to examine the simple main effects of NF training and Training stage to decompose the significant NF training ×Training stage interaction. A simple main effects test for Training stage showed that the MMN amplitudes significantly increased over time for groups that underwent NF training with genuine feedback (F(2, 54) = 5.81, p < 0.01), but not for groups in NF control condition with false feedback (F(2, 54) = 0.43, n.s.). We further analyzed this simple main effect of Training stage by conducting multiple comparisons using the Least Significant Difference method (MSe = 0.0096, p < 0.05) to compare each of the training days. The result showed a significant increase on the 3^rd^ training day compared with the 1^st^ training day (Fig 6). Fig 7 shows the group grand average MMN responses on each training day in the four groups, respectively. Due to the peak latencies were different for each participant, the result shown in Fig 7 differs somewhat from Fig 6. However, this figure also shows an increase in amplitude for groups that underwent NF training with genuine feedback, but not for groups in NF control condition with false feedback.

## Discussion

In this study, we designed and administered a gamified and adapted NF training to deliver MMN NF training to increase the ability of native Japanese speakers to distinguish between “light” and “right” in English. We compared NF training and general behavioral training in terms of behavioral performance and neural activity. First, we conducted a pre-training test prior to training to quantitatively evaluate the ability of Japanese monolingual participants to distinguish the English words “light” and “right.” Then, we divided the participants into four groups: the NTBT group, NTBC group, NCBT group, and NCBC group. After the training phase, we observed a significant improvement in the discrimination of “light” and “right” in all groups except for the NCBC group, and the improvement in the NTBT group was significantly higher than that in the other groups. Moreover, we found significant increases in neural activity in the groups that underwent NF training (NTBT and NTBC groups) but not in the NF control groups (NCBT and NCBC groups).

In this experiment, we designed and implemented a NF training game with three different scenarios, which were selected by the experimenter. In the game, the participant’s MMN amplitude was presented as visual feedback regarding discrimination accuracy, providing a continuous cue for learning. Although the participants were asked to ignore the auditory stimuli during the BCI game, there is a possibility that learning occurred unconsciously because they heard the stimuli repeatedly. However, the results in the NCBC group eliminated this possibility. Although the visual and auditory stimuli were the same in the NTBC and NCBC groups during the NF training games, we found no improvements in behavioral performance or neural activity in the NCBC group. These results suggest that the repeated stimuli did not improve auditory discriminability, and thus that the improvements in auditory discriminability were caused by genuine feedback in the NF training games.

A previous study showed that auditory recognition of /l/ and /r/ was improved following behavioral training [6]. In that study, participants pressed a button to identify a spoken word, and received feedback regarding whether their response was correct. However, this previous study did not show whether the auditory learning was caused by auditory stimuli or feedback. In the present study, we used a behavioral training method similar to that used in the previous study. However, during behavioral training in the present study, the participants in the BT group received real-time feedback, while the participants in the NCBC group did not. Our results also showed that repeated stimuli alone could not improve auditory discriminability. Thus, feedback during training is necessary, regardless of the form.

In terms of behavioral improvements, after completing all of the training sessions over 3 days, the participants in all groups except the NCBC group showed significant improvements. We also observed no significant differences between the NTBC group and NCBT group. Participants in the NCBT group received only general behavioral training, in which they had to consciously focus on listening and distinguishing the sound stimuli. In contrast, the participants in the NTBC group received only NF training. They did not have to focus on the sound stimuli, but their neural activity changed while playing the NF training game, and the sound discrimination training took place unconsciously [18, 23]. Although we found no statistically significant differences in behavior improvement between the two training methods, we found significant improvements in terms of neural activity in the groups that underwent NF training but not in the NF control groups. This suggests that NF training is more effective than behavioral training in changing neural activity. Some previous studies have associated increased brain activity with improved behavioral performance, even when participants are unaware that the training is taking place [18, 19, 22, 23]. They also indicated that auditory discriminability could be improved by solely increasing brain activity, even without behavioral discrimination training. This raises the possibility that NF training is more powerful than behavioral training, because NF could provide continuous feedback that accurately reflects the current state to induce learners to improve their brain processing and thus discrimination ability. In contrast, general behavioral training can only provide binary feedback (correct/incorrect) for responses. Because these responses are not necessarily identical to the subliminal brain responses that occur when a participant is unaware of the differences, binary feedback may be out of sync with the brain state and thus adversely affect learning.

Moreover, the NTBT group, which received both NF training and behavioral training, showed the greatest improvement, and this improvement was significantly greater than that in the other three groups. This result suggests that the combined use of the two training methods had a synergistic effect on auditory discrimination training.

At the end of the experiment, we interviewed all the participants. When asked what they thought about the game training experience, most participants answered, “The variety of the games is good,” and “the current games are satisfactory.” Some participants expressed their preference for one of the game scenarios, for example, “The Frisbee game was the most fun”; “I liked the paper toss game.” However, a few participants also stated that they felt bored during the trials and that they would have liked more game variety. This suggests that adding more variation to the game could alleviate participants’ boredom during training. In this study, to prevent excessive eye movements during the game, we designed the games in which the player aimed at a target point. For this reason, there are very few games to choose from, which greatly limits the variety of games and situations used for training, and thus the entertainment of training is limited. We plan to overcome this difficulty to provide more game variations for learners to choose from and increase the entertainment of training games in the future.

## Conclusion

In the present study, we developed a gamified and adapted NF training in the form of game scenario. We then compared the learning effect of NF training to that of general behavioral training in terms of the discrimination of English words. The results of the behavioral test showed that both general behavioral training and NF training significantly improved discrimination performance, and that the group exposed to both training methods had a significantly higher degree of improvement than the other groups. Furthermore, the NF training was more effective than the general behavioral training in enhancing brain activity. These results indicate that the strongest learning effect occurred when both methods were used together, and that combining the two training methods had a synergistic effect. Therefore, we suggest that NF training can be used with general behavioral training to enhance language learning.

## Supporting information

S1 Data (XLSX)
